# Evaluating the efficacy of treatment options for anal intraepithelial neoplasia: a systematic review

**DOI:** 10.1007/s00384-020-03740-6

**Published:** 2020-09-26

**Authors:** Danielle R. L. Brogden, Una Walsh, Gianluca Pellino, Christos Kontovounisios, Paris Tekkis, Sarah C. Mills

**Affiliations:** 1 Chelsea and Westminster Hospitals, NHS Foundation Trust and Imperial College, London, UK; 2 Colorectal Surgery, Vall d’Hebron University Hospital, Barcelona, Spain; 3 Department of Advanced Medical and Surgical Sciences, Università degli Studi della Campania Luigi Vanvitelli, Naples, Italy

**Keywords:** HIV, Cancer, Anal intraepithelial neoplasia, AIN, Anal squamous cell carcinoma

## Abstract

**Purpose:**

Anal intraepithelial neoplasia (AIN) is the accepted precursor of anal squamous cell carcinoma (ASCC). There has long been a hypothesis that treating AIN may prevent ASCC. Many different treatment modalities have been suggested and studied. We conducted this systematic review to evaluate their efficacy and the evidence as to whether we can prevent ASCC by treating AIN.

**Methods:**

MEDLINE and EMBASE were electronically searched using relevant search terms. All studies investigating the use of a single treatment for AIN that reported at least one end outcome such as partial or complete response to treatment, recurrence after treatment and/or ASCC diagnosis after treatment were included.

**Results:**

Thirty studies were included in the systematic review investigating 10 treatment modalities: 5% imiquimod, 5-fluorouracil, cidofovir, trichloroacetic acid, electrocautery, surgical excision, infrared coagulation, radiofrequency ablation, photodynamic therapy and HPV vaccination. All treatment modalities demonstrated some initial regression of AIN after treatment; however, recurrence rates were high especially in HIV-positive patients. Many of the studies suffered from significant bias which prevented direct comparison.

**Conclusions:**

Although the theory persists that by inducing the regression of AIN, we may be able to reduce the risk of ASCC, there was no clinical evidence within the literature advocating that treating AIN does prevent ASCC.

## Introduction

Anal intraepithelial neoplasia (AIN) is the accepted precursor to anal squamous cell carcinoma (ASCC). AIN and ASCC are due to dysplastic changes linked to human papillomavirus (HPV) infection, namely HPV 16, 18 and 31. There are several different classification systems for describing AIN, one, typically used in the UK, uses 3 stages of dysplasia, ranging from low-grade AIN1 to high-grade AIN3 before the development of ASCC. Another describes AIN as either “high-grade AIN” (HGAIN) or “low-grade AIN” (LGAIN). However, in 2012, the Lower Anogenital Squamous Terminology standardisation guidelines recommended the use of low- or high-grade “squamous intraepithelial lesion” (LSIL and HSIL), and many clinical centres have now adopted this as current practice [1].

By preventing HPV infection, it is thought that ASCC could become a preventable cancer; this is the rationale for adolescent HPV vaccination.

Patients with HIV represent an important high-risk group, especially in patients also reporting receptive anal intercourse, where the prevalence is reported to be as high as 87% in men [2] and 68% in women [3]. For this reason, much of the current evidence is derived from this population. Up to a third of patients in high-risk groups have high-grade AIN or ASCC [2, 3]. However, the rate of progression to ASCC from AIN is uncertain; Fuchs et al. (2016) states that up to a third of patients with high-grade AIN develop ASCC within 3 years [4], whereas other studies suggest that it is a much rarer occurrence, 7.4 cases per 100 years of follow-up [5].

There exists a hypothesis that by treating AIN in high-risk groups, we may prevent the development of ASCC. However, there is no consensus on which modality of AIN treatment is optimum and whether treatment for AIN is efficacious in preventing ASCC. Most treatments also have significant side effects that limit their acceptability for long-term use.

Many varied approaches and modalities are used to treat AIN. These include topical treatments, electrocautery, excision and/or destruction of lesion, laser ablation, photodynamic therapy, argon plasma coagulation, radiofrequency ablation and infrared coagulation. Topical treatments such as 5% imiquimod, 5-flurorouracil (5-FU) and 1% cidofovir are some of the most studied.

With the many different treatment modalities used for AIN, it is unsurprising that little consensus exists in guidance for best clinical practice. We present a systematic review of all available AIN treatments in the literature with the aim of informing best practice guidance resulting in a more uniform approach to AIN treatment.

## Method

This is a systematic review performed following the Preferred Reporting Items for Systematic Reviews and Meta-Analyses (PRISMA) Statement [6]. The systematic review protocol has been submitted to the International Prospective Register of Systematic Reviews (PROSPERO ID CRD42019135487).

### Inclusion criteria

All studies investigating the use of a single treatment for AIN in any patients with a prior histological diagnosis of low-grade or high-grade AIN were included. To be included the papers required at least one of the end outcomes (partial or complete response to treatment, recurrence after treatment or ASCC diagnosis after treatment) to be reported. Any relevant peer-reviewed case studies were included alongside comparative studies as there is little evidence currently on the best modality of AIN therapy and there would be a risk of bias towards more studied modalities if smaller studies in novel techniques were excluded.

### Exclusion criteria

We excluded non-human studies and studies that were not available in the English language. We also excluded studies that did not meet the inclusion criteria, did not report any required end outcomes or studies that solely included patients with anal warts or patients without an established histological diagnosis of AIN. Single case reports and non-peer-reviewed abstracts and letters were excluded.

### Data sources and search strategies

A MEDLINE and EMBASE search from inception to 18 April 2020 was conducted using the search strategy (“Anal Intraepithelial Neoplasia” OR “AIN” OR “LSIL” OR “HSIL” OR “LGAIN” OR “HGAIN”) AND (“treatment” OR “therapy” OR “management”). Reference lists of included studies were also reviewed as well as current clinical guidelines and registered clinical trial registers. The titles and abstracts obtained from the electronic search were systematically reviewed against the inclusion criteria for relevant full papers to be obtained and read. On reading full papers, if they met the required inclusion criteria, they were included in the review. Duplicate papers were excluded on abstract review. DRLB performed the original data search as well as data extraction. UW performed a second independent data extraction for accuracy and also performed an independent check of abstract and full-paper review against inclusion criteria to ensure investigator agreement. To allow a fair comparison between studies, data included in systematic review was by default expressed as “intention to treat”.

### Outcome definitions

“Complete response” is defined as high- or low-grade AIN that on follow-up after treatment no AIN of any grade persists.

“Partial response” is defined as patients with high-grade AIN that after treatment have residual low-grade AIN only on follow-up.

“Recurrence” is defined as any grade of AIN that recurs at any grade after a previous complete response to AIN treatment or recurs at high-grade if there was a partial response to treatment on follow-up. When it was possible to separate patients with synchronous untreated lesions on follow-up from the reported recurrence rate, we did so to allow a fair comparison. This is clearly indicated on Table 1.

**Table 1:** Topical AIN treatments

Study	Design	*N*	Mean age	Male(%)	HIV-positive (%)	MSM(%)	High-gradeAIN (%)	Compliance(%)	CR (%)	PR (%)	Recurred (%)	ASCC(%)	Follow-up(median inmonths)	Level ofevidence	Bias score
Imiquimod 5%: treated with cream or suppository 3 times a week × 16 weeks															
Weilandet al.2006 [9]	Prospectivenon-randomisedopen-label -pilot study	28	43	100	100	100	64 (46)	79	54	17.9	16	0	9	4	Moderate +
Kreuteret al.2008 [10]	Prospective follow-up study	19	-	100	100	100	68	100	74	-	26 at treatedsite (58 atuntreated site)	0	32	4	Serious +
Fox 2010et al. [11]	Double-blind RCT	53	42	100	100	100	100	83	14	29	39	2 (placebo arm)	33	1b	Low*
Richel et al.2013 [12]	Open-label RCT	54	45 (median)	100	100	100	57	91	24 (16 HGAIN)	11	71	0	4.5 (response) 16.5 (recurrence)	1b	Some concerns*
Cranstonet al.2018* [13]	Prospective,non-randomisedopen-label pilotstudy	10	46 (median)	100	100	100	100	90	30		-	0	-	4	Moderate +
*N* = 164															
1% Cidofovir: [23] 2 g of self-applied cream three times a week for 4 weeks; [22] self-applied cream six 2-week treatment cycles (5 days ontreatment 9days off treatment)															
Sendagortaet al.2016 [20]	Prospective,non-randomisedpilot study	17	36	100	94	100	100	94	59	18	12	0	5.5	4	Moderate +
Stier et al.2013 [19]	Phase 2a prospectivemulticentre trialopen-label	33	44	73	100	-	100	79	15	36	-	3	1.4	4	Moderate +
*N* = 50															
5-Fluorouracil:16 weeks of treatment, 0.25 to 1 g self-administered, Synder et al 2011 treatment duration 9 weeks only															
Grahamet al. 2005 [15]	Prospective,non-randomisedopen-label pilotstudy	7	48	45	9	-	100	100	86	0	0	0	39 (mean)	4	Serious +
Richel et al.2010 [14]	Prospective,non-randomisedopen-label pilotstudy	46	46 (median)	100	100	100	74	93	39	17	50	0		4	Moderate +
Snyder et al.2011* (remove star[16]	Retrospective single-intervention case review at a single center	11	45 (median)	100	100	100	82	100	9	27	-	0	-	4	Moderate +
Richel et al.2013 [12]	Open-label RCT	48	47 (median)	100	100	100	60	96	17 (21 HGAIN)	12.5 (21 HGAIN)	58	0	4.5 (response) 16.5 (recurrence)	1b	Some concerns*
*N* = 112															
80% Trichloroacetic acid - Cranston et al. 2014 - 5 Q-tips worth of TCA applied under direct vision at HRA × 1; Singh et al. 2019 - up to 4 applications of TCA (1–2 month intervals) on direct vision at HRA															
Cranston et al.2014 [21]	Retrospectivechart review	72	48	100	100	-	100	100	72	11	15 at index site(22.6 at indexand adjacent32 synchronous)	-	-	4	Serious +
Singh et al.2009 [22]	Retrospectivechart review	54	44 HIV-positive;45 HIV-negative	100	65	-	52		28	14	28	-	12	4	Moderate +
*N* = 50															

+Robins I tool *Cochrane riskof bias tool

“Incidence of ASCC” is defined as any new diagnosis of ASCC on follow-up after previously treated AIN. Patients with a previous history on ASCC before treatment for AIN are excluded.

### Levels of evidence, quality and bias assessment

Studies will be assessed and classified by the Oxford Centre for Evidence-Based Medicine Levels of Evidence 2011 [7].

A bias assessment will also be undertaken in all included studies. Randomised controlled trials will be assessed using the Cochrane Risk of Bias Version 2 Tool [8] and non-randomised studies using the ROBINS-I tool [9].

## Results

### Description of studies

**Fig. 1:**
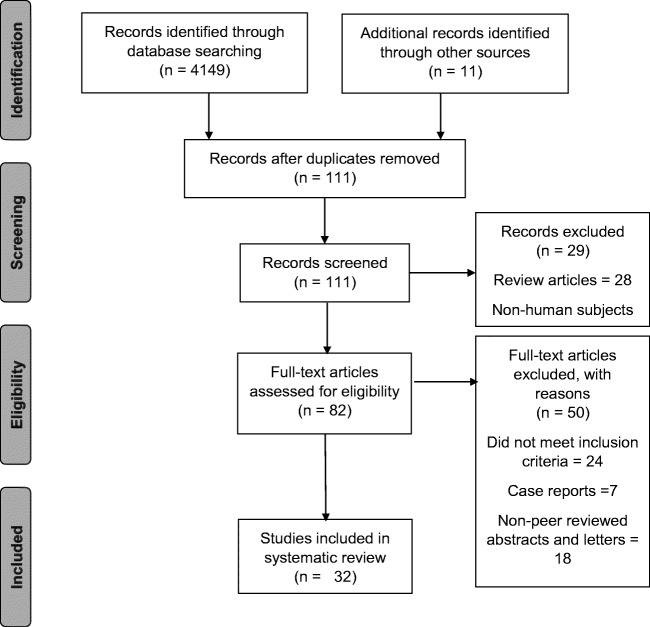
PRISMA Table—results of the search strategy and reasons for exclusion

A total of 4149 studies were identified after an electronic search of MEDLINE and EMBASE, 100 studies underwent abstract review and 82 studies underwent full-text review. Rationale for exclusion of studies is included in Fig. 1. Thirty-two studies were eventually included in the systematic review which included outcomes from imiquimod, 5-fluorouracil, cidofovir, trichloroacetic acid, electrocautery, surgical excision, infrared coagulation, radiofrequency ablation, photodynamic therapy and HPV vaccination. Unfortunately, argon plasma coagulation and laser ablation could not be included in the analysis as either only non-peer-reviewed abstracts were identified or the outcomes of single treatment modalities within the study were not reported.

A total of 26 included papers were classified as level 4 in the Oxford Centre for Evidence-Based Medicine Levels of Evidence 2011 and only 4 randomised controlled trials were identified.

A significant number of papers that were identified were published prior to the LAST criteria guidance therefore did not express their results as either HSIL or LSIL. To make a fair comparison, we will define “high-grade AIN” as either HGAIN, HSIL, AIN2 or AIN3 and “low-grade AIN” as LGAIN, LSIL or AIN1.

### Imiquimod

5% Imiquimod is a Toll-like Receptor 7 (TRL7) agonist that binds to TRL7 and activates the production of cytokines involved in the Th1 pathway, an immune response for virus-infected cells [10]. It is currently not licenced for use in treating AIN and is used off label. Its side effects include pain and bleeding on the area undergoing treatment which affects treatment compliance. Kreuter et al. (2007) demonstrated that levels of p16, a protein associated with HPV viral DNA, reduce with the use of imiquimod [11], so there is evidence that imiquimod does act on HPV-infected dysplastic cells, and could be a viable treatment for AIN.

Five studies were found which met the inclusion criteria (*N* = 164) [12–16]. All reported the response rate of imiquimod in HIV-positive “men who have sex with men” (MSM). Study participants had between 14 and 86% total response to treatment and 5–35% partial response to treatment.

Despite many studies reporting local self-limiting adverse events such as bleeding, pain and anal irritation, overall compliance with treatment was good ranging from 79 to 100%. Non-compliance when reported was more likely due to the study protocol and requirements on participants rather than adverse events.

There was one randomised double-blind controlled trial comparing the use of imiquimod against placebo; this however only showed 14% complete response and 29% partial response with a 39% recurrence of high-grade AIN over median of 36 months in HIV-positive MSM [14]. This trial included a further 4 months of treatment for those in the treatment group who failed to respond and a crossover methodology to allow patients in the placebo arm access to imiquimod treatment.

There was also another randomised controlled trial that compared the use of imiquimod, 5-flurouracil and electrocautery in HIV-positive MSM. In this trial, Richel et al. (2013) showed that when treated with imiquimod, 24% of study participants had a complete response and 11% had a partial response. However, the participants also had a very high recurrence rate (71% of patients that achieved 72 weeks follow-up) [15].

It is also worth noting that a study which conducted a subset analysis of compliant patients only found a much higher complete response rate of 74% and a further 5% with a partial response [13].

The results suggest, although there appears to be an initial response to the imiquimod treatment, recurrence is high. The quality of evidence was also low with most studies reporting high level of bias. There is no evidence that imiquimod alone prevents the progression of AIN to ASCC, and due to the high levels of recurrence of high-grade AIN after treatment, it is unlikely that there is any benefit treating HIV-positive MSM. Particularly as there is a paucity for evidence for repeated treatment and compliance could be problematic. There were no included studies investigating the use of imiquimod in HIV-negative patients or HIV-positive women.

### 5-Fluorouracil

5-FU is a pyrimidine analogue that inhibits thymidylate synthase which is involved in DNA synthesis [17]. It is licenced for use topically in basal cell carcinomas and actinic keratosis but is used off licence for the treatment of HPV-related intraepithelial neoplasia [17]. Similarly to imiquimod, side effects such as pain, discomfort and bleeding do affect treatment compliance.

Four studies examined the use of 5-fluorouracil in the treatment of AIN [18, 17, 15, 19]. Three studies included HIV-positive MSM as their only participants [17, 20, 19].

Study participants had between 9 and 86% complete response and 0–27% partial response. Recurrence rates ranged from 9 to 58%, and there were no new cases of ASCC reported in follow-up periods. The recurrence rate in HIV-positive MSM was between 0 and 58%.

Richel et al. (2010) showed that the participants that did respond to treatment did have a significant reduction in HPV 16 DNA load, but 85% of participants experienced side effects from the treatment such as anal pain and proctitis, and the frequency of which 48% was significant [17].

There was one randomised controlled trial comparing 5-fluorouracil with imiquimod and electrocautery in HIV-positive MSM [15]. Richel et al. (2013) showed that after treatment with 5-flurouracil, high-grade AIN completely responded in 21% and partially responded in 21%, but 58% recurred over the follow-up period of 72 weeks [15].

Like imiquimod, it appears that 5-fluorouracil does lead to some regression of AIN but with a greater variance in successful treatment. There was a significant rate of recurrence in HIV-positive MSM; however, when comparing like for like such as in Richel et al. (2013), the recurrence rate was less than that of imiquimod. More studies are required to assess its use in women and HIV-negative patients.

### Cidofovir

One percent cidofovir is a cytidine nucleotide analogue which has shown to treat HPV in vitro [21]. It has been used successfully to treat anal warts, and pilot studies exist evaluating its use in AIN [22].

Two studies met the inclusion criteria, both studied the use of cidofovir on HIV-positive men and women. Sendagorta et al. (2016) reported 59% had complete response with 18% recurrence rate [23]. In Stier et al. (2013), 15% of patients with high-grade AIN had a complete response and 36% had a partial response; however, the follow-up was very short (6 weeks) and 97% of patients reported an adverse side effect and 21% of participants did not complete the treatment course due to severe side effects. In addition, progressive disease to ASCC was seen in 3%.

Although treatment with cidofovir did lead to regression of AIN, there is currently insufficient evidence that cidofovir could be of benefit in preventing ASCC, as reported side effects may make long-term use unsustainable. The recurrence rates, when stated, in HIV-positive individuals were lower than other topical treatments.

### Trichloroacetic acid

Trichloroacetic acid is a topical treatment that had been used successfully in the treatment of anal warts [24]. It is corrosive and is used to remove the top layer of treated skin. Like the other topical treatments, it is associated with side effects such as pain, bleeding and discomfort at the treated site.

Two studies met the inclusion criteria; Cranston et al. (2014) used 80% trichloroacetic acid in HIV-positive men with high-grade AIN, 72% had either a complete response and 11% had a partial response with a 15% recurrence rate in 3–6 months of follow-up [25].

Singh et al. (2009) used 85% trichloroacetic acid in HIV-positive men and women with AIN. Twenty-eight percent had a complete response and 15% had a partial response. Twenty-eight percent of patients treated had a recurrence of AIN (72% of patients who were compete responders) [24].

Although trichloroacetic acid does appear to regress AIN, there is insufficient evidence about its side effect profile or its long-term efficacy in the treatment of AIN.

### Surgical excision

Surgical excision historically used to be a much more widely used practice due to the lack of other treatments available, and the hypothesis that full thickness excision prevented recurrence. This has now largely fallen out of fashion due to the increased risk of anal stenosis and poor sexual function. Therefore, only two historical papers could be included in this review; Scholefield et al. (1994) demonstrated a 30% recurrence of high-grade AIN after wide local excision, whereas Brown et al. (1999) demonstrated an 18% had a recurrence rate. It is also important to note that this study reported that only 14% of patients achieved a complete excision without AIN identified on the margins [26].

### Electrocautery

Electrocautery and surgical destruction are when suspicious lesions are cauterised using diathermy by the surgical or sexual health teams. There are risks of complications and side effects including pain, bleeding, synchronous lesions, anal stenosis and infection [27, 28].

Six studies met inclusion criteria [29, 28, 15, 30–32], 2 of which included HIV-positive MSM only [31, 30, 15, 28].

Complete response to electrocautery was between 22 and 78% and 2 studies reported that partial response occurred in between 7 and 26% of HIV-positive patients with high-grade AIN [31, 15].

Interestingly, two studies reported outcomes from HIV-negative patients; Marks et al. (2012) reported that 85% of HIV-negative patients had a complete response to electrocautery and were statistically less likely recur when compared with HIV-positive patients [32]. In Chang et al.’s study (2002), there were no HIV-negative patients that recurred after treatment [29].

Burgos et al. (2016) also identified that 39% of patients treated had a recurrence at the previously untreated site and that high recurrence rates could also be related to metachronous disease [31].

Electrocautery is less well-tolerated in terms of sexual function and quality of life at 20 weeks after treatment when compared with imiquimod and 5-FU [33].

Like topical treatments, electrocautery appears to have some effect in treating AIN, however, with a high recurrence rate particularly in HIV-positive patients. Electrocautery may be more successful in HIV-negative individuals.

The majority of cauterisations are performed under local anaesthetic in clinic [31] which raises the question whether this technique would be more successful if performed under sedation or under general anaesthetic allowing optimal views and access to lesions.

### Infrared coagulation

Infrared coagulation (IRC) is a newer technique that although is associated with pain and bleeding, the symptoms are reported to be less severe than other ablative treatments (Table 2) [27]. It involves applying a probe that directs short bursts of infrared light to areas of concern [25].

**Table 2:** Ablative treatments

Study	Design	*N*	Mean age	Male (%)	HIV-positive (%)	MSM (%)	High-grade AIN (%)	Treatment length	CR (%)	PR (%)	Recurred(%)	ASCC(%)	Follow-up (median in months)	Level of evidence	Bias score
Electrocautery: HRA-guided destruction of AIN lesions															
Chang et al. 2002 [26]	Prospective non-randomised open-label pilot study	37	45	100	78	65	100	-	22	0	-	-	32.3 in HIV-negative and28.6 in HIV-positive(mean)	4	Moderate +
Pineda et al. 2008 [25]	Retrospectivechart review	246	44	84	74	-	100	One or multipletreatments ifcircumferential	78	-	57	0.4	42	4	Critical +
Marks et al. 2012 [29]	Retrospective chart review	232	43 HIV-negative, 49 HIV-positive (median)	-	57	100	100	Up to 4 treatmentswith 3–4 months follow-upperiods	67 (after4th treatment)	0	-	0.4	17.3 in HIV-negative,13 inHIV-positive after 1st treatment	4	Moderate +
Richel et al. 2013 [12]	Open-labelRCT	46	47 (median)	100	100	100	54	4 months	39 (40HGAIN)	7 (12 HGAIN)	28	0	4.5 (response)16.5 (recurrence)	1b	Some concerns *
Assoumou et al. 2013 [27]	Retrospective chart review	80	42 (median)	100	56	100	90	1 treatment	53	-	-	0	-	4	Critical +
Burgos et al. 2016 [28]	Observationalcohort study	108	-	100	100	100	100	2–4 treatmentsfollowed byHRA 6–8 weekslater. This wasrepeated untilclearance.	25	26	13	0	12 (mean)	4	Moderate +
*N* = 749															
Infrared coagulation: after HRA, 1.5–1.6 second pulses under direct vision															
Cranston et al. 2008 [32]	Retrospectivechart review	68	45 (median)	100	100	100	97 (lesions)	1 treatment	11	66	-	0	4.6 (mean)	4	Moderate +
Stier et al. 2008 [36]		18	44	89	100	-	44 (lesions)	1 treatment butretreated at 3 months ifpersistentdisease	39	17	11	0	12	4	Moderate +
Goldstone et al. 2011 [33]	Retrospectivecohort	143	42 HIV-positive; 36 HIV-negative	100	48	100	67	Up to 4treatments	45 (after 4thtreatment)	-	-	0	69 HIV-positive; 48HIV-negative	4	Serious +
Weis et al. 2012 [31]	Prospectivecohort	124	40	80	100	-	100	1 treatment	3	69	-	0	33	2B	Serious +
Sirera et al. 2013 [34]	Retrospectivecohort	69	43	45	100	74	100	1 treatment, further treatment if recurrence	71	6	-	0	25 (mean)	4	Moderate +
Goldstone et al. 2019 [35]	Non-blinded multisiterandomised controltrial (IRC vsobservation alone)	120	49 treatedvs. 50.5untreated	90 treated 97 vs. untreated	100	-	100	1–3 treatments depending on response	62 treated vs. 30 untreated	7 treated vs. 6 untreated	-	0 (both arms)	12	1b	Some concerns*
*N* = 542															
Radiofrequency ablation: intravenous sedation and local anesthetic, 3 applications of energy at 12 J/cm^2^															
Goldstone et al. 2017 [37]	Prospective,non-randomisedopen-label pilot study;hemi-circumferentialRFA	21	45	86	0	-	100	1 treatment. Further treatment if recurrence	52	-	14	0	12	4	Moderate +
Goldstone et al. 2017[38]	Prospective,non-randomisedopen-label pilotstudy: circumferentialRFA	10	52	100	90	-	100	1 treatment. Further treatment if recurrence	60	0	0	0	12	4	Moderate +
*N* = 31															

+ Robins I tool* Cochrane risk of bias tool

Six studies were found in the literature all with varying response rates; 3–71% of patients had a complete response, whereas 6–69% of patients had a partial response [34–37]. Interestingly, Goldstone et al. (2011) reported recurrence rates which were considerably higher in HIV-positive MSM when compared with that of HIV-negative MSM; however, the majority of this burden of recurrence was caused by metachronous lesions rather than recurrence of lesions recently treated by infrared coagulation [36].

One randomised controlled trial was identified where HIV-positive patients with HSIL were randomised to either receive IRC or routine observation with high-resolution anoscopy [38]. The results in this trial were promising; 71% of patients in the treatment arm were dysplasia-free at 12 months compared with 28% in the active observation arm (*p* < 0.001). The trial itself appeared to be well-conducted and only limited by its inability to blind patients to the intervention received. There were no new cancers identified in either treatment arm, but the study was underpowered for this outcome.

Two patients in the placebo arm of one study developed ASCC on follow-up; overall they demonstrated that infrared coagulation did result in a reduction in high-grade disease with ASCC on follow-up; however, despite this, they did not separately analyse risk of ASCC between study arms. It is unlikely, due to small study numbers, that this would be statistically significant [34].

### Radiofrequency ablation

More frequently used in endoscopy units to treat dysplasia is related to Barrett’s oesophagus; Radiofrequency ablation uses multiple electrodes to provide radiofrequency energy to cause the coagulation of tissue at high temperatures.

Radiofrequency ablation (RFA) as yet has only been reported in the literature by one author. Goldstone et al. (2017) reported their outcomes of hemi-circumferential [39] as well as circumferential RFA for high-grade AIN [40].

In one study, Goldstone et al. (2017) performed hemi-circumferential RFA in HIV-negative patients with high-grade AIN and reported 14% recurrence after 1 year in RFA-treated areas. In the other study, Goldstone et al. (2017) performed circumferential RFA in patients with high-grade AIN (90% were HIV-positive). On the first treatment, 40% of patients had persistent lesions; however, after a further treatment, they achieved a 0% recurrence rate at 1 year follow-up in 10 patients.

Although this sounds promising as a treatment modality, more research needs to be completed to be able to comment with confidence on the use of RFA for high-grade AIN.

### Photodynamic therapy

Photodynamic therapy involves the ablation of AIN by applying light sources to areas previously photosensitized [41]. Photosentisation can be undertaken systemically, by the IV administration of a photosensitising agent, or topically by the application of the agent on the area of concern [41]. Van der Snoek et al. (2012) advises that the advantage of using photodynamic therapy is that light can be applied more evenly with higher precision than other ablative methods [41].

Two studies were identified that met inclusion criteria; Van der Snook et al. (2012) used systemic photodynamic therapy to treat HIV-positive MSM with high-grade AIN. Twenty percent had a complete response to treatment and 27% had a partial response. There was a 20% recurrence rate and 6% of patients had a worsening of dysplasia during treatment [41].

Welbourn et al. (2014) also reported outcomes of topical, systemic, and a combined treatment on 13 patients and demonstrated a 40% complete response. However, there was little information on study participants; therefore, it is unclear whether the population included in the study is generalisable to other studies [42].

### HPV vaccination

The quadrivalent HPV 6, 11, 16 and 18 vaccine (Gardasil, Merck) has been proposed as a method of secondary prevention for the recurrence of high-grade AIN. This is based on studies such as Palefsky et al. (2006) that demonstrated 33% complete or partial response to an experimental quadrivalent vaccination in high-grade AIN [43].

More recently, Swedish et al. (2012) have shown that the quadrivalent vaccination prevents the recurrence of high-grade AIN after targeted destruction [44]. However, this is contrary to a randomised controlled trial of patients with high-grade AIN that received the quadrivalent vaccine or a placebo vaccine. Unfortunately, the trial was stopped early due to finding no benefit between its treatment arms after median follow-up of 3.4 years [45]. It is therefore unlikely that HPV vaccination is an effective treatment for high-grade AIN.

## Discussion

This systematic review has identified several treatment modalities that do induce regression of AIN; however, the majority of the studies were underpowered to identify whether there is any evidence that treating AIN could prevent the development of ASCC in the long term. Furthermore, as the recurrence rates are high, and treatment are often associated with poor compliance due to severe side effects, it is possible that treating AIN could result in more harm than overall benefit (Tables 1, 2 and 3).

**Table 3:** Other treatment modalities

Study	Design	*N*	Mean age	Male (%)	HIV-positive (%)	MSM (%)	High-gradeAIN (%)	Treatmentlength	CR (%)	PR (%)	Recurred (%)	ASCC (%)	Follow-up (median in months)	Level of evidence	Bias score
Surgical excision															
Scholefieldet al. 1994[41]	Retrospective chartreview	27	45	-	-	-	100	-	-	-	30	11	20 (mean)	4	Critical +
Brown et al.1999 [23]	Retrospective chartreview	34	44	-	-	-	100		-	-	18	-	41	4	Critical +
*N* = 61															
Photodynamic therapy															
Van de Snoeket al. 2012 [39]	Prospective open-label pilot study	15	46	100	100	100	100	1 systemictreatment	20	27	20	0	-	4	Serious +
Welbournet al. 2011[40]	Retrospective chartreview	15	52	47	-	-	-	1 systemic ortopicaltreatment	40	20	20	0	19	4	Serious +
*N* = 30															
HPV vaccination															
Swedishet al. 2012[42]	Prospective cohort	202	40	100	0	100	100	3 doses ofquadrivalentHPVvaccination	-	-	13vaccinated 31unvaccinated	0	60 unvaccinated 41 vaccinated	2B	Moderate +
Wilkin et al.2018 [43]	Double-blindRandomisedControlled Trial	575	47 vaccinated 48 placebo	82	100	-	67	3 doses ofquadrivalentHPVvaccination	-	-	16vaccinated 16placebo	0	41	1C	Low*
Palefskyet al. 2006[44]	Phase 1 + 2 trial ofexperimentalHPV vaccineSGN00101	15	48	87	100	-	100	3 doses ofexperimentalHPV vaccineSGN00101	7	27	0	0	11	2B	High*
*N* = 31															

+ Robins I tool* Cochrane risk of bias tool

The majority of studies identified to be included in his systematic review were also of poor quality and likely to lend to bias. For example, many studies were inconsistent in how they defined high-grade AIN and not all studies followed up all patients and reported their outcomes in an “intention to treat” manner. Some studies were also limited in power due to their sample sizes and length of follow-up time. For this reason, we have chosen not to undertake a meta-analysis of the results.

There was also some difficulty identifying comparable recurrence rates between studies. Not all studies reported whether their recurrences were at a treated site or whether they developed another focus of dysplasia at a synchronous or metachronous site. The high recurrence rates could easily be driven by a continuing HPV perineal infection. Better study methodology is required to be able to determine whether treatment of each individual lesions is efficacious. A whole-field approach like the pilot study completed by Goldstone et al. (2017) which treated the entire anal squamocolumnar junction with radiofrequency ablation, if well-tolerated long-term, may be the best option for the future treatment of high-grade AIN.

Only four randomised controlled trials were identified, and unfortunately, they all described different treatments: one examining the use of imiquimod compared with placebo [14], one comparing quadrivalent HPV vaccination with placebo [45], a further trails comparing infrared coagulation against active monitoring [38] and a three-armed trial by Richel et al. (2013) comparing imiquimod, 5-fluorouracil and electrocautery [15]. The different treatment modalities in each trial meant their results could not be compared directly with each other. All four trials were of good quality however; therefore, their findings are most likely to be accurate. On balance, the clinical trial recommendations on the topical treatments for AIN in HIV-positive MSM are similar; Fox et al. (2010) reported 43% response to imiquimod treatment compared with that of Richel et al. (2013) who had a 46% response. The recurrence rates were not similar however 39% compared with 71% respectively [14, 15].

Goldstone et al. (2019) demonstrated very promising results with infrared coagulation achieving statistically higher rates of complete response compared with active monitoring alone with 71% of treated patients being disease-free at 12 months [38]. Their treatment protocol was quite comprehensive, with 45% of treated patients receiving 2 or 3 treatments over the study period. It may be that to achieve a good outcome with ablative therapies that multiple treatments may be required in the long term. It would be interesting to see whether patients who were disease-free at the end of the study remained so in the long term or whether they will later require further ablative therapies in the future.

Richel et al.’s study (2013) was the only study identified that compared multiple treatments; they demonstrated a significantly higher complete response rate in electrocautery compared with 5-fluorouracil (*p* = 0.008). Electrocautery also resulted in a higher complete response rate compared with imiquimod, but this was not statistically significant (39% vs. 24%, *p* = 0.10) [15]. There was no statistically significant difference in recurrence rate between treatment modalities [15].

We are aware that some clinical centres use topical treatments as an adjunct to ablative therapies; however, the evaluation of this treatment pathway is beyond the scope of this review which is limited to the study of single treatment modalities. It is possible that a combination of treatment modalities may have a better long-term result, and further studies should be undertaken with this in mind.

These findings correspond with recent guidelines published. The Association of Coloproctology of Great Britain and Ireland suggests that HIV-positive MSM would most likely benefit from electrocautery but did not state a specific preference on topical treatments [46], whereas The American Society of Colon and Rectal Surgeons also advises no preference between ablative treatments, imiquimod, 5-fluorouracil, trichloroacetic acid and cidofovir for the treatment of low- and high-grade dysplasias [47]. The Italian Society of Colorectal Surgery recommends that topical treatments could be a good compromise between surgical treatment and the watch and wait approach. They feel strongly that a method of treatment should be undertaken for all high-grade disease but like the American and British guidelines expressed no other preference on the best treatment modality [48].

The majority of the available evidence is based on HIV-positive MSM, which lends to the possibility that different treatments may be more successful in HIV-negative populations. Indeed, several studies that did include a comparison between populations suggested that HIV-negative patients had a lower recurrence rate [36, 32, 29] More research is required in comparing different patient subpopulations with AIN.

There also exists a high possibility of publication bias as many single centres are reporting their outcomes in non-peer-reviewed abstracts that could not be included in this analysis. Other centres are reporting novel techniques such as Goldstone et al.’s (2017) use of radiofrequency ablation. As there is no other corresponding research in other centres into this treatment modality, although they demonstrated good outcomes, generalisability is potentially limited.

In 2014, the Anal Cancer HSIL Outcomes Research (ANCHOR) study, a multicentre stage III randomised controlled trial began recruiting HIV-positive patients in the USA (NCT02135419). The study is randomising patients with a new diagnosis of high-grade AIN to receive either ablative treatment, topical treatment or watchful waiting with time to ASCC incidence as the trial’s primary outcome measure. It is hoped that this trial will be able to clarify whether there are long-term benefits to treating HIV-positive patients with high-grade AIN.

## Conclusion

There is no single treatment modality that has a good enough evidence base to recommend its use as the gold standard in the treatment of AIN. Nearly all of the studies included in this review were able to demonstrate AIN regression, but recurrence rates were often high and the evaluation of the long-term efficacy of the treatments was limited by short follow-up times. Although the theory persists that by inducing the regression of AIN, we may be able to reduce the risk of ASCC, there was no clinical evidence within the literature advocating that treating AIN does prevent ASCC. Further clinical trials are required at a larger scale with longer follow-up times that include HIV-negative as well as HIV-positive patients.

## Data Availability

Not applicable.
